# Fabrication of vertical GaN/InGaN heterostructure nanowires using Ni-Au bi-metal catalysts

**DOI:** 10.1186/1556-276X-8-299

**Published:** 2013-06-26

**Authors:** Ryong Ha, Sung-Wook Kim, Heon-Jin Choi

**Affiliations:** 1 Department of Materials Science and Engineering, Yonsei University, Seoul 120-749, South Korea

**Keywords:** Coaxial heterostructure nanowires, Longitudinal heterostructure nanowires, Gallium nitride

## Abstract

We have fabricated the vertically aligned coaxial or longitudinal heterostructure GaN/InGaN nanowires. The GaN nanowires are first vertically grown by vapor–liquid-solid mechanism using Au/Ni bi-metal catalysts. The GaN nanowires are single crystal grown in the [0001] direction, with a length and diameter of 1 to 10 μm and 100 nm, respectively. The vertical GaN/InGaN coaxial heterostructure nanowires (COHN) are then fabricated by the subsequent deposition of 2 nm of In_x_Ga_1-x_N shell on the surface of GaN nanowires. The vertical GaN/InGaN longitudinal heterostructure nanowires (LOHN) are also fabricated by subsequent growth of an InGaN layer on the vertically aligned GaN nanowires using the catalyst. The photoluminescence from the COHN and LOHN indicates that the optical properties of GaN nanowires can be tuned by the formation of a coaxial or longitudinal InGaN layer. Our study demonstrates that the bi-metal catalysts are useful for growing vertical as well as heterostructure GaN nanowires. These vertically aligned GaN/InGaN heterostructure nanowires may be useful for the development of high-performance optoelectronic devices.

## Background

Gallium nitride (GaN) is a promising material for optoelectric and electronic devices such as laser diodes, light-emitting diodes, solar cells, and high-performance field effect transistors [1,2] Meanwhile, nanowires have been of great interest as building blocks for high-performance nanodevices because of their high crystalline quality, large surface-to-volume ratio, and size confinement effects. Accordingly, GaN nanowires have great potential for application in high-performance optoelectronics [3]–[5].

The growth of GaN nanowires have been discussed in many previous studies [2,6,7]. The modulation of nanowires, for example, the preparation of a vertical array, creation of a heterostructure, and doping, has also been studied to exploit the potential of nanowires. One of the issues in this modulation is the fabrication of vertically aligned nanowires because it is necessary for the manufacturing of optical nanowire devices with high performance [4,8]–[11]. Compared to randomly oriented nanowires, vertically aligned nanowires have a specific growth orientation and uniformity in their height and diameter. Owing to these properties, nanowire devices can be easily manufactured using the vertical semiconductor integration scheme. The optical properties of these devices can be optimized by their well-defined nanowire orientation, size uniformity, and well-ordered structures [4,8,11,12]. For example, the vertical nanowire array can provide stimulation emission as well as 0% reflectance of incident light, which are critical for the development of high-performance lasers and solar cells, respectively [8,9]. The other issue in the modulation of nanowires is the fabrication of heterostructure nanowires such as coaxial heterostructure nanowires (COHN) or longitudinal heterostructure nanowires (LOHN) that can tune and maximize optoelectronic properties. For example, the luminescence from the GaN/InGaN COHN can be tuned for the entire visible light wavelength (1.12 to 3.34 eV) on the basis of the In composition in the InGaN shells [13]. The InGaN shell in the COHN is also helpful in achieving efficient radiative recombination of injected carriers, while confining both carriers and photons in the nanowires.

Nanowires are grown by means of a vapor–liquid-solid (VLS) mechanism [14]. This mechanism can be used to grow nanowires vertically by establishing an epitaxial relationship between the nanowires and substrates [15]–[21]. In the case of GaN nanowires, however, vertical growth using the VLS mechanism has rarely been reported [22]. This is because an interfacial layer is formed on the substrates by the vapor-solid (VS) mechanism prior to the growth of GaN nanowires by the VLS mechanism, thus preventing the establishment of an epitaxial relationship between nanowires and substrates [23]. It is thus difficult to grow vertically aligned GaN nanowires reliably using the current VLS mechanism.

In this report, we present a method to grow GaN nanowires vertically via the VLS mechanism using Au/Ni bi-metal catalysts. We also demonstrate the fabrication of GaN/InGaN COHNs or LOHNs using these vertically grown GaN nanowires and the tunability of the optical properties of the nanowires.

## Methods

GaN nanowires were grown by means of metal organic chemical vapor deposition using trimethylgallium (TMGa) and ammonia (NH_3_) as group III and V precursors, respectively. Nickel/gold thin films (0.5/2-nm thick) were deposited on the sapphire (c-Al_2_0_3_) substrate coated with a 3-nm-thick GaN film (c-plane). Homemade reactor, consisted with furnace (Model Blue M, Lindberg Co., Ltd., Asheville, NC, USA) and quartz tube with diameter of 1 inch, was used for the growth of GaN nanowires. The substrates were loaded into a quartz reactor and heated to the growth temperature (800°C) for 25 min under the flow condition of 100 sccm H2 and 100 sccm N2. The GaN nanowire was grown at 800°C for 30 min by flowing 0.5 sccm of TMGa and 50 sccm of NH_3_ and then cooled down to room temperature.

The GaN/InGaN COHNs were fabricated on a vertically grown GaN nanowire by further depositing the InGaN and GaN shell on the surface of the nanowire at 600°C to 750°C using TMGa, TMIn, and NH_3_. InGaN LOHNs were also fabricated on a vertically grown GaN nanowire by further supplying TMGa and TMIn and NH_3_ to the catalyst. The InGaN layer was grown at 550°C. The nanowires were characterized using scanning emission microscopy (SEM), transmission emission microscopy (TEM), and energy-dispersive spectroscopy (EDS). The optical properties of fabricated nanowires were investigated using photoluminescence (PL). Micro-PL was used to characterize the optical properties of the LOHN.

## Results and discussion

**Figure 1:**
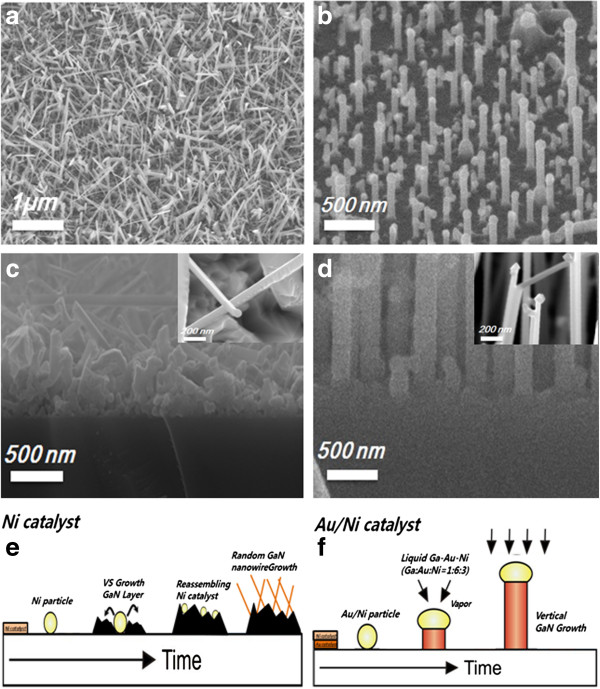
**SEM images of GaN nanowires grown by the vapor**–**liquid**-**solid mechanism. (a)** SEM images of GaN nanowires grown by Ni catalysts. **(b)** SEM images of GaN nanowires grown by Au/Ni catalysts. **(c)** Cross-sectional SEM images of GaN nanowires grown by Ni catalysts. Inset of **(c)** shows the end of the nanowires. **(d)** Cross-sectional SEM images of GaN nanowires grown by Au/Ni catalysts. Inset of **(d)** shows the end of the nanowires. **(e)** Schematic illustration of the VLS process for GaN nanowire grown by Ni catalysts. **(f)** Schematic illustration of the VLS process for GaN nanowire grown by Au/Ni catalysts.

Figure 1a shows a typical SEM image of the GaN nanowires grown on the substrate using Ni as a catalyst. Ni is a well-known catalyst for the growth of GaN nanowires [24]. However, the nanowires grow randomly on the substrate. In fact, the vertical growth of GaN nanowires has rarely been achieved using a Ni catalyst.

Figure 1c is the SEM image of the nanowire-substrate interface. It can be seen that the substrate is covered by an interfacial layer on which GaN nanowires grow randomly. The inset of Figure 1c shows the end of the nanowires. A metal globule can be observed at the end, which clearly indicates that the nanowires are grown by the VLS mechanism. The diameter and length of nanowires are 80 to 100 nm and several hundred micrometers, respectively. Because the nanowires grow on the interfacial layer, the interfacial layer is grown prior to the nanowires, though the catalyst for the nanowires is coated on the substrate. This means that the VS mechanism of direct deposition of GaN from the vapor for the growth of the interfacial layer works at the early stage, prior to the working of the VLS mechanism. Previous reports have shown that the initial GaN grows on the interfacial layer after the GaN nanowires are grown using Ni catalyst [23]. It was found that the catalyst does not work in the early stage, in which the interfacial layer instead grows on the substrate due to a VS mechanism. After the catalyst works, the GaN nanowires grow on the interfacial layer due to a VLS mechanism. The Ni catalyst, leading to the VLS process of nanowires in the second step is reassembled from the metal films onto the surface of the interfacial layers [23]. Therefore, the growth of the interfacial layer is expected to be faster than that of the nanowires in the case of the Ni catalyst.

This may result from the complexity of the VLS mechanism. The VLS mechanism involves three phases and two interfaces (specifically, vapor–liquid and liquid–solid interfaces). The chemical reactions of dissolution and precipitation are involved in the working of the VLS mechanism, which is not the case with the VS mechanism [25]–[27]. Diffusion in the gas and liquid phases is also involved. These reactions and diffusions during the VLS mechanism require high activation energies. Meanwhile, the growth of nanowires via the VLS mechanism competes with the counter growth of interfacial thin layer via the VS mechanism. Generally, the VS mechanism is simple as compared to the VLS mechanism, which involves three phases and two interfaces [26,27]. Thus, the activation energy for the VS mechanism is lower than that for the VLS mechanism and thus could initiate earlier. This interfacial layer interrupts the epitaxial relationship between the nanowires and the substrate, as this layer is polycrystalline and thus has a surface with various crystalline directions. This results in the random growth of GaN nanowires, as shown in Figure 1a.

Figure 1b shows the nanowires grown by Au-Ni bi-metal catalysts. It shows the vertical growth of nanowires. Figure 1d shows the interfaces between the nanowires and the substrate without the interfacial layer. That is, the GaN nanowires grow directly from the substrate. The result indicates that Au has a critical role in preventing the formation of the interfacial layer, thereby enabling the epitaxial vertical growth of GaN nanowires. The inset of Figure 1d shows the end of nanowires grown by the Au/Ni catalyst. It shows the metal globule at the end of nanowires and clearly indicates that the nanowires are grown by the catalyst via VLS mechanism. The diameter and length of nanowires were 80 to 100 nm and several hundred micrometers, respectively.

One of the possible explanations of the role of Au in the vertical growth of nanowires is its ability to lower the liquid formation temperature as well as the activation energy of the VLS mechanism that leads to the growth of nanowires on the substrate prior to the deposition of the interfacial layer. It is well known that the liquidus temperature of the multicomponent metal system decreases with the number of components. In this regard, the addition of Au to Ni should decrease the liquidus temperature of the Au-Ni-Ga system as compared to that of the Ni-Ga system and can thus lead to the growth of nanowires via the VLS mechanism at low temperature, prior to the VS deposition of the interfacial layer [23,25]. Based on these results, the growth processes of random growth and vertical growth GaN nanowires can be outlined in Figure 1e, f, respectively. In the case of random growth, the GaN interfacial layers are first deposited on the substrate, after which, the catalyst is reassembled on the interfacial layer; finally, the GaN nanowires randomly grow on the interfacial layer by the VLS mechanism. In the case of vertical growth, the Au/Ni catalyst works before the deposition of the interfacial layer, and the GaN nanowires vertically grow on the substrate.

**Figure 2:**
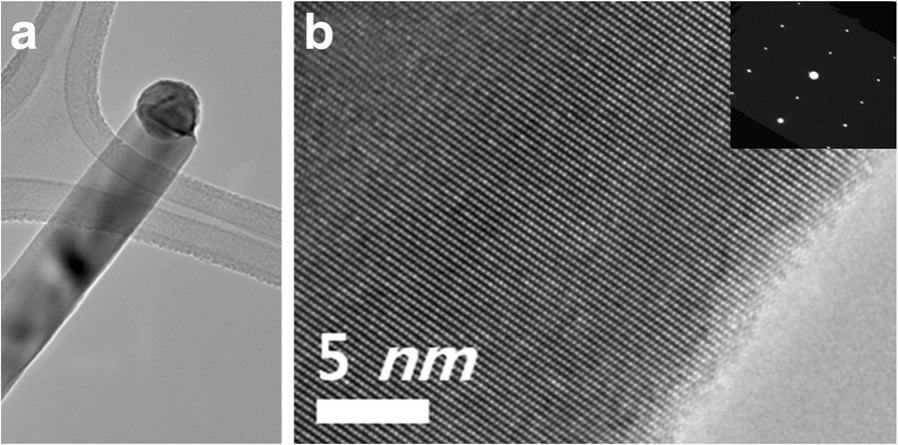
**A typical TEM image. (a)** Low-magnitude TEM image and **(b)** HRTEM image of a GaN nanowire grown by Au/Ni catalysts. The inset SAED pattern in **(b)** shows that the direction of GaN nanowire was [0001].

Figure 2a, b shows the TEM images of an individual nanowire. The TEM analysis also shows that the nanowires are single crystalline without defects. High-resolution TEM observation and selected area electron diffraction (SAED) analysis, recorded along the [2-1-10] zone axis, clearly show that the nanowires are single crystalline. X-ray diffraction (XRD) was used to determine the crystal structure of GaN nanowires. Two XRD peaks of (0002) and (0004) in the XRD pattern indicate that GaN nanowires have wurtzite structure [16] (Additional file 1: Figure S1).

In this study, the vertical growth of GaN nanowires has been successfully achieved. The technique used would be helpful for the fabrication of nanowire devices with high-performance optical properties, using semiconducting processes. Higher performance optical properties can be expected when a COHN or LOHN is achieved in these vertical nanowires. For example, the luminescence can be improved by creating a GaN/InGaN COHN with a luminescence that is tunable by the composition of the InGaN layer and a large surface area that extends along the entire length of the nanowires with carrier separation in the radial direction [13]. To explore this potential, the COHN is fabricated using vertical GaN nanowires.

**Figure 3:**
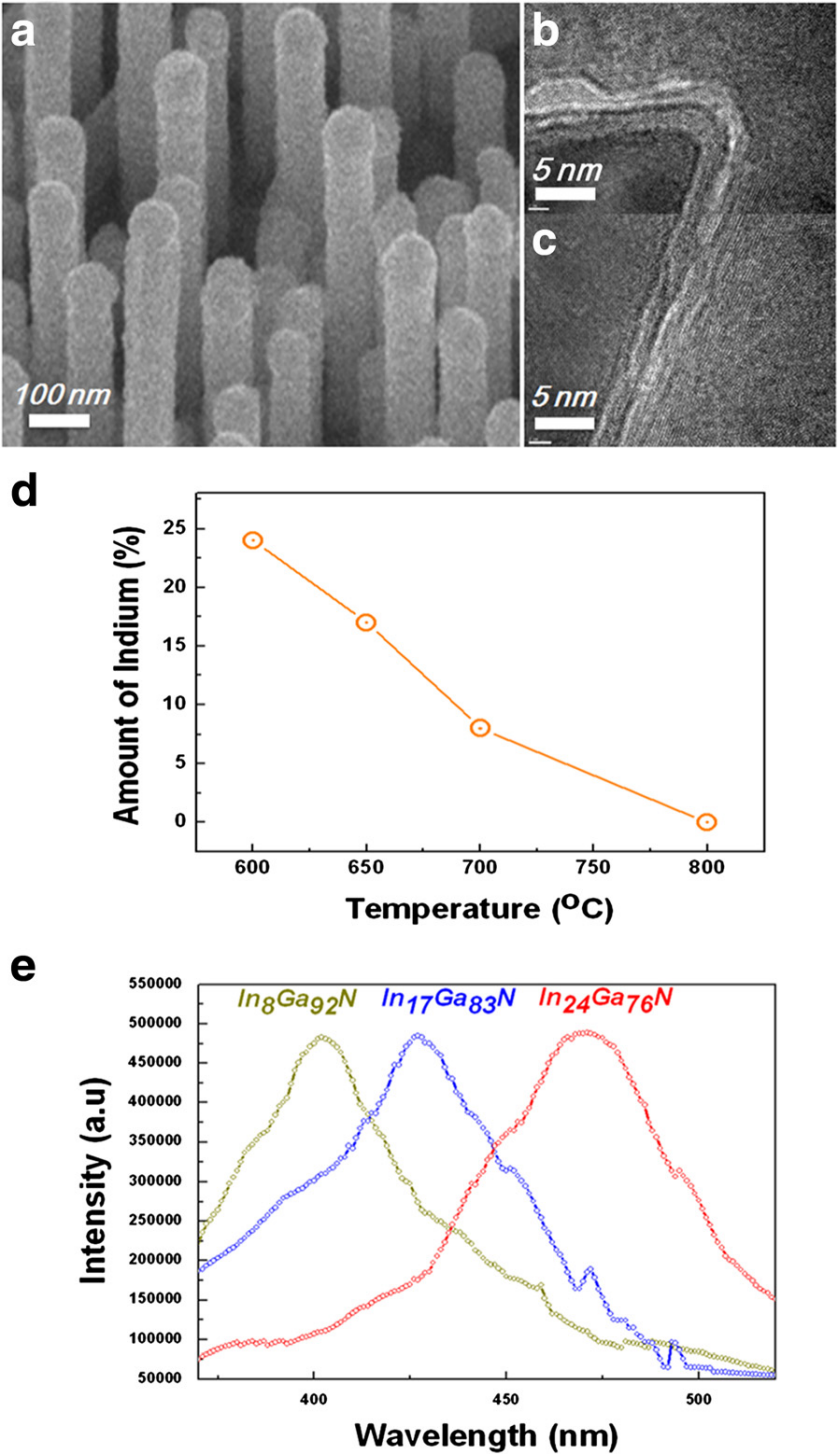
**The GaN**/**In**_**x**_**Ga**_**1**-**x**_**N COHN. (a)** SEM images of COHN nanowires. **(b)** Cross-sectional TEM images of corner area of COHN nanowire. **(c)** Cross-sectional TEM images of flat area of COHN nanowire **(d)** The indium composition in InGaN shells as a function of growth temperature. **(e)** The normalized PL spectra of COHN grown at 600°C to 750°C.

Figure 3a shows the SEM image of a COHN prepared by the deposition of InGaN and GaN layers on the GaN nanowires. As shown in the figure, the prepared nanowires have a larger diameter than the GaN nanowires due to the deposition of InGaN/GaN layer on the outer surfaces. Figure 3b,c shows the cross section of the COHN. As shown in the figure, the nanowire has a triangle shape [13]. Figure 3b shows the corner side of nanowire and Figure 3c shows the flat side of nanowire, respectively. It shows that InGaN and GaN shell are deposited homogeneously at both corner and flat sides. It is composed of the GaN core region, InGaN shell in the middle, and GaN shell at the surface. The diameter and thickness of the inner GaN core region, outer InGaN shell, and GaN shell are, 80 to 100 nm, 2 nm, and 2 nm, respectively. The thickness of the shells could be controlled by the deposition time in our CVD systems.

The In composition of InGaN shell could also be adjusted. According to the previous study, the In compositions of this shell are affected by the growth temperature. Generally, the amount of In is gradually depleted with the increase in temperature [13,28] because TMIn, which is the precursor for In, easily decomposes as compared to TMGa and is, thus, sensitive to the temperature. We studied the relationship between the growth temperature and the In concentration in the InGaN layers in our CVD system. For this study, the InGaN shell is deposited to a thickness of 50 nm on the GaN nanowires for the convenience of the compositional analysis of the shell by TEM since the typical InGaN shell, which is several nanometers thick, is difficult to analyze. Figure 3d shows the In composition in InGaN shells as a function of temperature. It shows that the amount of In has a linear relationship with the temperature and that In is gradually depleted with the increase in temperature. An EDS was used to determine the composition in the InGaN shell (Additional file 2: Figure S2).

The optical properties of a vertical COHN (with 2-nm-thick InGaN and 2-nm-thick GaN shells) were characterized through excitation by a He-Cd laser (wavelength of 325 nm) and subsequent measurement of the PL. Figure 3e shows the normalized PL spectra of COHN grown at 600°C to 750°C. COHN shows wavelengths ranging from violet to light green. The peak, the center of PL wavelengths, shifts to longer wavelengths from 405 to 425 and 475 nm (3.06, 2.92, and 2.61 eV in photon energy) as indium concentration increases [13,28]–[30]. This indicates that the optical properties of vertical COHNs can be tuned on the basis of the composition of the InGaN shell.

**Figure 4:**
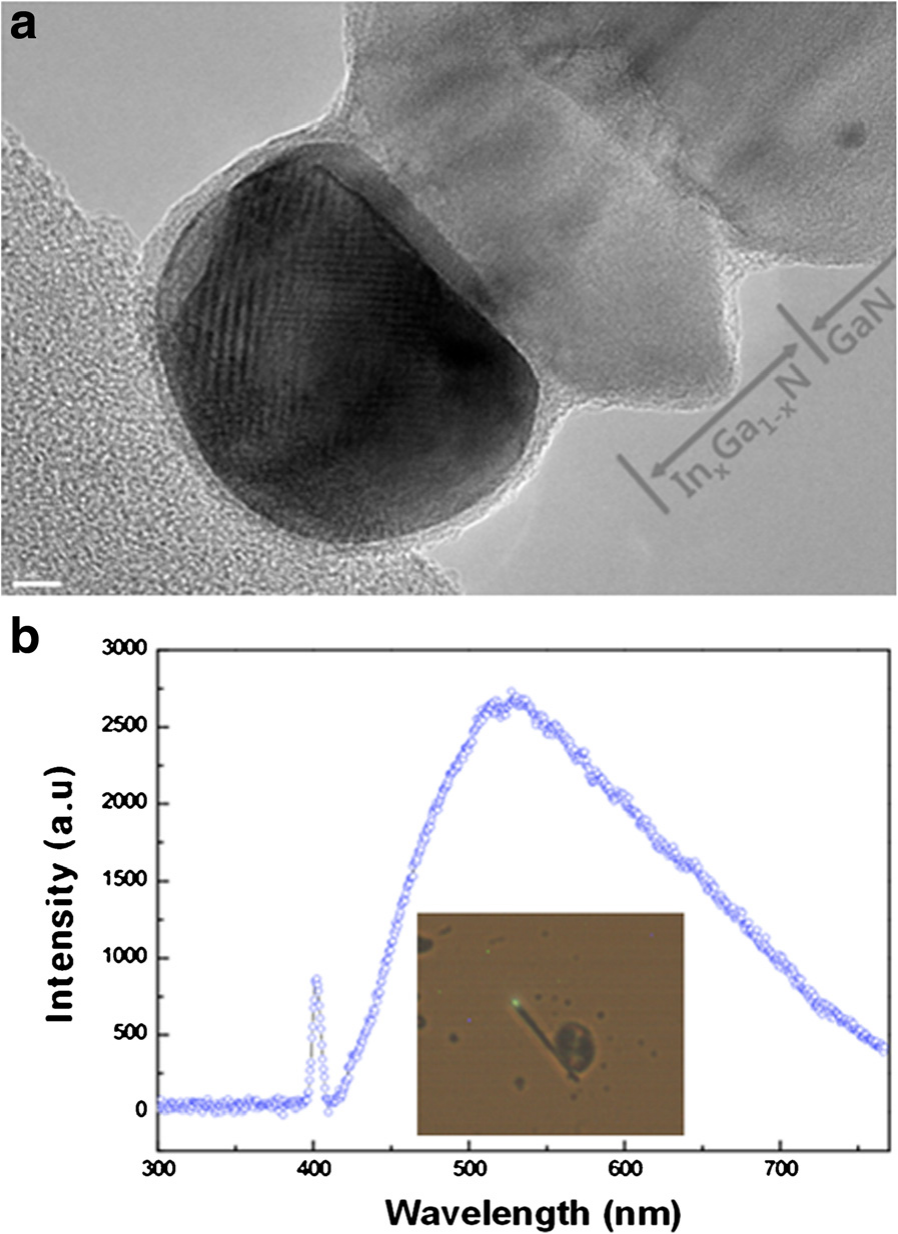
**The GaN**/**In**_**x**_**Ga**_**1**-**x**_**N LOHN. (a)** TEM images of LOHN nanowires. **(b)** Micro-PL of the individual LOHN nanowire. Inset of **(b)** shows the green emission of end of the LOHN nanowires.

LOHNs can also provide improved optical properties of GaN nanowires. For example, LOHN serves the quantum structures in a longitudinal direction, which enhances the optical properties due to the quantum confinement effect [13,31]. The PL and electroluminescence can also be improved by creating an LOHN p-n junction. To explore these potentials, we have fabricated the vertical LOHN, based on vertical GaN nanowires. Figure 4a shows the GaN/In_x_Ga_1-x_N LOHN. Our study indicates that the LOHN can be prepared at a lower temperature (for example, 550°C) compared to that for COHN (600°C to 800°C) under the same conditions. This lower temperature may due to the early liquefying of the bi-metal catalysts and the dissolution of the Ga and In precursors at low temperature, prior to the deposition of the shell on the side surface of the nanowires by the VS mechanism. Hence, the vertical LOHN as well as COHN can be fabricated in our system by simply controlling the processing temperature. The TEM image shows two layers with the metal catalyst. According to our compositional analysis, the bright layer close to the metal catalyst is the 5-nm-thick In_0.4_Ga_0.6_N layer and below that is the pure GaN layer.

In the COHN, the growth of the InGaN layer on the GaN nanowires proceeds through the VS mechanism. However, in the LOHN case, the growth of the InGaN layer proceeds through the VLS mechanism via a catalyst. This difference results in a compositional difference in the heterostructures. For example, the InGaN layers constitute 40% of the LOHN whereas they constitute 25% of the COHN, under the same processing conditions except for a temperature difference of 50°C. By considering the temperature differences of 50°C, this compositional difference (i.e., indium rich in the layer in LOHN formed by VLS mechanism) is still significant that may come from the different growth mechanism [31]. The higher In composition in the VLS mechanism may be due to the precipitation of the InGaN phase from the thermodynamically supersaturated In-Ga-Ni-Au liquid phase that has a higher In/Ga ratio than the atmosphere. Our analysis shows that the composition of the metal catalyst of In-Ga-Ni-Au is *ca*. 20%, 10%, 20%, and 50%, respectively, when prepared under the same ratio of TMIn and TMGa in the atmosphere. This indicates that the InGaN layer in the LOHN is different from that in the COHN because it shifts to the indium-rich sides, owing to the indium-rich supersaturated composition of the liquid metal catalyst. Although only one composition is reported here, our further study shows that the composition of the InGaN layers grown by VLS mechanism via a catalyst can be controlled by the processing temperature from 0% to 50%. This indicates that the composition of the InGaN layer in LOHN can also be tuned easily by the processing temperature. Figure 4b shows the micro-PL of the individual nanowire of the GaN/In_0.4_Ga_0.6_N LOHN. A green emission can be seen at the InGaN layer with a wavelength of 520 nm. It indicates that the optical properties of the vertical GaN nanowires can be tuned by fabricating the LOHN by a VLS mechanism via bi-metal catalysts.

## Conclusions

In summary, we have achieved the vertical growth of GaN nanowires via a VLS mechanism using Au/Ni bi-metal catalysts, which leads to the growth of nanowires without the interfacial layer between the nanowires and the substrate and, in turn, enables their vertical growth. TEM studies have shown that the GaN nanowires are single-crystalline and dislocation-free. The vertical GaN/InGaN COHN can then be fabricated by subsequent deposition of In_x_Ga_1-x_N shell onto the GaN nanowires. The vertical GaN/InGaN LOHN can also be fabricated by the subsequent growth of an InGaN layer using the catalyst. These outcomes demonstrate that bi-metal catalysts are versatile for the vertically aligned as well as the heterostructure GaN nanowires. Optical studies of the COHN and LOHN have demonstrated InGaN composition-dependant emission from 405 to 520 nm. Vertically aligned GaN and heterostructure nanowires (COHN, LOHN) with tunable optical properties can be expected to be useful for the fabrication of high-performance optoelectronic devices.

## Competing interests

The authors declare that they have no competing interests.

## Authors’ contributions

RH carried out the experiment and drafted the manuscript. SWK and HJC participated in the design of the study and drafted the manuscript. All authors read and approved the final manuscript.

## Supplementary Material

Additional file 1: Figure S1 Two XRD peaks of (0002) and (0004) in the XRD pattern indicate that GaN nanowires have wurtzite structure [16]. Click here for file

Additional file 2: Figure S2 An EDS was used to determine the composition in the InGaN shell. Click here for file
