# Supplementary figures and images for: Fabrication of vertical GaN/InGaN heterostructure nanowires using Ni-Au bi-metal catalysts
Source: Nanoscale Res Lett. 2013 Jun 26;8(1):299. doi: 10.1186/1556-276X-8-299 (PMC3720202; doi:10.1186/1556-276X-8-299)

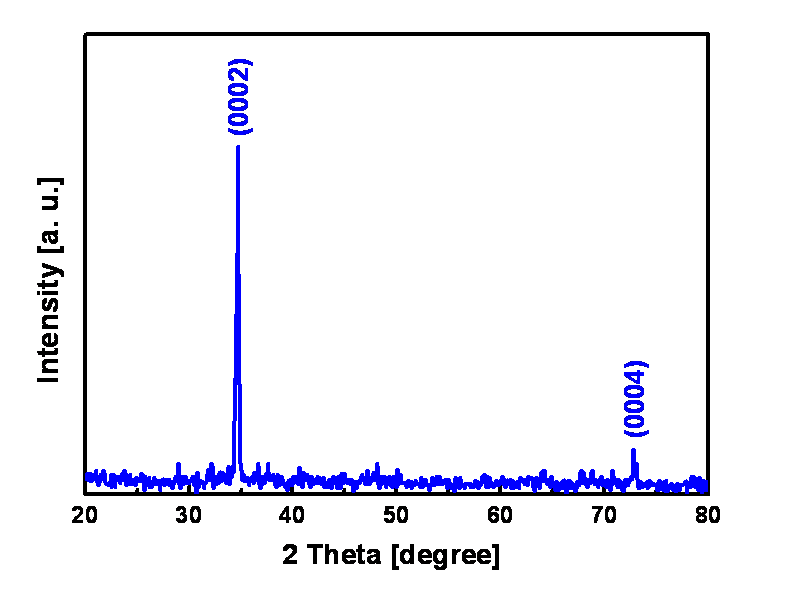

Supplement: Additional file 1: Figure S1 — Two XRD peaks of (0002) and (0004) in the XRD pattern indicate that GaN nanowires have wurtzite structure [16]. [file 1556-276X-8-299-S1.tiff]

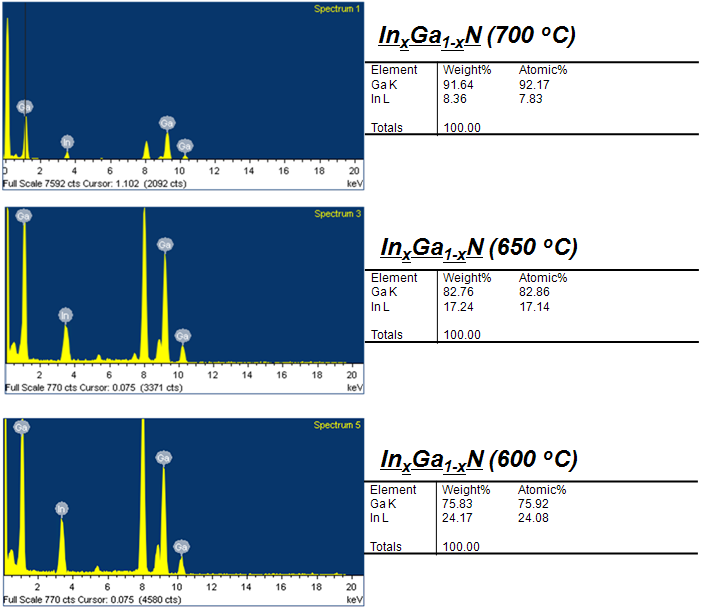

Supplement: Additional file 2: Figure S2 — An EDS was used to determine the composition in the InGaN shell. [file 1556-276X-8-299-S2.tiff]
